# Structure, development, and the salt response of salt bladders in *Chenopodium album* L.

**DOI:** 10.3389/fpls.2022.989946

**Published:** 2022-09-08

**Authors:** Yigong Zhang, Ayibaiheremu Mutailifu, Haiyan Lan

**Affiliations:** Xinjiang Key Laboratory of Biological Resources and Genetic Engineering, College of Life Science and Technology, Xinjiang University, Urumqi, China

**Keywords:** *Chenopodium album*, halophyte, salt bladder, polysaccharides, NaCl

## Abstract

Salt bladders are specialized epidermal structures that halophytes use to store and excrete excess salt. However, the cell wall composition during salt bladder development is unclear, and the functions of salt bladders in a few wild plants remain unexplored. Therefore, the present study examined salt bladder development, cell wall composition, and their roles under salt stress by employing bladder-brushed and unbrushed *Chenopodium album* plants. We found that the bladder cell of *C. album* was connected to the epidermal cells through a rectangular stalk cell and developed from the shoot tip and the young leaves. The polysaccharides of salt bladder cell wall showed dynamic distribution at different stages of development. Moreover, salt bladders affected Na^+^ and K^+^ accumulation, increased reactive oxygen species scavenging, and improved the osmoregulation and photosynthetic efficiency in leaves, subsequently enhancing the salt tolerance of plants. The findings strengthen our knowledge of the physiological mechanisms of the accessory structures in desert plants, which can be used as a reference for further research at the molecular level.

## Introduction

Soil salinization is a major issue that inhibits plant growth and development, limiting agricultural production worldwide (Zhao et al., 2020). Most crops are sensitive to salt stress. Nevertheless, few plants, called halophytes, are salt-resistant or salt-tolerant and can live in saline environments for a long time. These plants have evolved unique salt tolerance mechanisms to cope with specific environmental stress (Flowers and Colmer, 2008). Approximately 50% of halophytes cope with salt stress through specialized epidermal structures called the epidermal bladder cells or the salt bladders, which are often the modified form of trichomes and accumulate and excrete excess salt *via* rupture (Ding et al., 2010; Shabala and Mackay, 2011). Thus, salt bladders play an important role in stress tolerance *via* salt secretion, water storage, and osmotic regulation (Shabala et al., 2014).

Salt bladder cells have a huge central vacuole as the primary salt storage site and a dense cytoplasm that contains a nucleus, mitochondria, chloroplasts, and other organelles; typically, the bladder cell remains connected to the leaf epidermal cells through stalk cells (Smaoui et al., 2011; Guo et al., 2020). Salt bladder cells, composed of stalk and bladder cells, originate from the plant epidermis. During bladder complex formation, the “destined” epidermal cell develops a dense protoplasm and protrudes perpendicular to form the initial cell of the salt bladder; subsequently, the protuberant cell divides into two cells, producing a stalk cell and a bladder cell (Shabala et al., 2014). As the bladder cell matures, it continuously absorb salt, swells, or breaks, releasing ions into the environment (Yuan et al., 2016). However, there are some exceptions, such as *Mesembryanthemum crystallinum*, which has no stalk cells; here, the salt bladder cells are directly connected to the epidermal cells (Barkla et al., 2012, 2016).

Salt stress drastically reduces the intracellular turgor pressure of plants, causing dehydration and shrinkage. However, halophytes accumulate sodium (Na^+^) and potassium (K^+^) ions and provide sufficient turgor pressure for cell morphological maintenance and volume expansion; these ions get secreted through a break by an external force (Shabala, 2013). Salt bladder formation has been associated with endopolyploidy. It has been proposed that increased ploidy helps mitigate stress damage, and the increase in cell size under salinity contributes to tolerance by expanding the store size for Na^+^ sequestration in *M. crystallinum* (Barkla et al., 2018). Moreover, salt bladders continuously absorb salts under salt stress, increasing intracellular turgor pressure. The changes in turgor pressure influence the reorientation of microtubules and actin cytoskeleton. Studies have also shown that the transcriptional level of V-ATPase in salt bladders significantly increased under salt stress. The V-ATPase helps in vacuolar Na^+^ uptake and turgor pressure generation, promoting the rapid expansion of bladders cells (Barkla et al., 2016).

In addition, salt bladders confer ion sequestration in the vacuole and protect plants from ionic poisoning. The bladder cells accommodate salts at a concentration 60 times higher than the mesophyll cells (Wu et al., 2018). In quinoa (*Chenopodium quinoa* Willd.), under salt stress, the salt bladders accumulated 50% K^+^ and 40% Na^+^ and chloride (Cl^−^), leading to robust salt tolerance; however, when the salt bladders were removed, a large amount of Na^+^ and Cl^−^ remained in the leaves, resulting in a salt-sensitive phenotype (Kiani-Pouya et al., 2019). In *Atriplex canescens*, brushing the salt bladders reduced the growth and photosynthetic indexes under salt stress; these brushed plants could not excrete salt, and a large amount of Na^+^ got accumulated in the mesophyll tissue, causing ionic toxicity and osmotic stress (Guo et al., 2019, 2020). Meanwhile, the bladderless mutant of *M. crystallinum* demonstrated reduced salt tolerance and productivity, with significantly lower Na^+^ and Cl^−^ content in the aerial parts than in the wild type (Agarie et al., 2007). Research has also shown that the salt bladders can accumulate osmotic regulators and metabolites and maintain intracellular ion balance and osmotic stability, thereby improving plant salt tolerance and maintaining normal growth and development (Barkla et al., 2016; Böhm et al., 2018). However, the changes in the cell wall composition during salt bladder development have not been analyzed. Moreover, the functions of the salt bladders have not been thoroughly investigated in some wild plant species.

*Chenopodium album* L. is an annual wild herb that grows in saline-alkali regions and has excellent drought and saline-alkali resistance (Yao et al., 2010b). Its seeds can achieve a high germination rate under 300 mM NaCl stress, and the plants can tolerate 300 mM NaCl treatment (Yao et al., 2010a). The leaves of *C. album* have a high density of salt bladders, which gradually break down and fall off with development. However, the physiological mechanisms *via* which salt bladders regulate the impact of Na^+^ in *C. album* remain unexplained. Therefore, the present study examined the biogenesis of salt bladders in *C. album* and analyzed their cell wall composition. Furthermore, we investigated the role of salt bladders in stress response by employing brushed (to remove salt bladder) and unbrushed *C. album* plants. The findings of this research will extend our understanding of the biogenesis and functions of salt bladders in desert plants.

## Materials and methods

### Plant growth conditions and NaCl treatment

*Chenopodium *album** seeds were obtained from Urumqi County in Xinjiang, China. These seeds were rinsed repeatedly with purified water and allowed to germinate in Petri dishes at 25°C in the dark for 7 days. Seedlings with uniform growth were transplanted into plastic pots (15 cm × 10 cm, five plants/pot) filled with a mixture of *nutrient soil, vermiculite, and perlite (2:1:1)* and maintained in a greenhouse at 25°C (day/night), 16 h/8 h photoperiod (light/dark; 800 μmol m^−2^ s^−1^ flux density), and 45% relative humidity. Plants were allowed to grow for 4 weeks and then treated with Hoagland solution containing salts as follows, maintaining 10 pots per treatment: (1) Control (Hoagland solution, 0 mM NaCl); (2) low salt stress (Hoagland solution with 100 mM NaCl); (3) high salt stress (Hoagland solution with 300 mM NaCl). After 7 days, the seedlings were collected to determine the physiological parameters.

### Analysis of epidermal cell development

The true leaf, young leaves, and mature leaves at the early stages of *C. album* growth were collected to analyze the epidermal cell development. The collected tissue was first fixed in 50% FAA (formalin-acetic acid-alcohol) solution at 4°C for 24 h and dehydrated at different concentrations of ethanol and xylene. The tissue was then embedded in paraffin for about 3 h at 60°C, and the paraffin blocks were sliced into 5–7 μm sections with a microtome (Leica RM2126, Leica, Germany). The tissue sections on the slides were deparaffinized and stained with 1% safranin overnight and then with 0.5% fast green for 3 s. The stained sections were sealed and observed under a microscope (SMZ25, Nikon, Japan).

### Analysis of cell wall composition of salt bladders

Immunocytochemistry was performed to determine the composition of the bladder cell wall. The tissue section on the slide was incubated in blocking solution (1% bovine serum albumin in phosphate buffer saline; pH 7.2; PBS-BSA) at 37°C for 1 h and then with diluted (1:10 in BSA) CCRC-M1 (recognizes fucose (Fuc)-containing epitope), CCRC-M7 (arabinogalactan epitope), and CCRC-M38 (de-esterified homogalacturonan) primary monoclonal antibodies (Carbosource Services, Georgia, United States) for 1 h. After incubation, the slides were washed five times with PBS-BSA and allowed to react with horseradish peroxidase (HRP)-labeled secondary antibody (goat anti-mouse IgG) diluted in PBS-BSA (1:500) at 37°C for 1 h. In control, the primary antibodies were replaced with PBS. Finally, all sections were stained with diaminobenzidine (DAB; ZSGB-BIO, ZLI-9018) for 3 min and dehydrated twice in absolute ethanol and xylene for 5 min. The sections were observed under a Nikon ECLIPSE Ti-E microscope, and the digital images were captured with a Nikon DS-Fi1 digital camera; the extended focal range images were analyzed and visualized with the NIS-Elements D imaging software (version 4.0).

### Determination of Na^+^ and K^+^ concentrations in leaves under salt stress

The leaves were used to analyze the Na^+^ and K^+^ concentrations after removing salt bladders from both sides of the leaf with a soft nylon brush, as described previously (Pan et al., 2016); the unbrushed leaves were used as the control. The Na^+^ and K^+^ ions were extracted from the oven-dried brushed and unbrushed tissues (young leaf and mature leaf) with 1% nitric acid under 120°C for 2 h and analyzed by inductively coupled plasma mass spectrometry (iCAP™ RQ ICP-MS, Thermo Fisher, United States), as described earlier (Cheng et al., 2004).

### Physiological analysis of leaves under salt stress

Seedlings were grown for 4 weeks under controlled conditions. One day before the commencement of salt stress, the salt bladders were gently brushed off from the leaf surface using a nylon brush. All salt bladders on the stem and petioles were also removed. Plants were then irrigated daily with 100 or 300 mM NaCl for 2 weeks. As new leaves emerged, salt bladders were regularly removed from the leaf surface and the petioles until the end of the experiment. Proline and betaine content, malondialdehyde (MDA) and hydrogen peroxide (H_2_O_2_) levels, and catalase (CAT), superoxide dismutase (SOD), and peroxidase (POD) activities in the leaves were measured following the microplate technique using the corresponding detection kits (G0111W, G0122W, G0109W, G0112W, G0105W, G0101W, and G0107W, respectively; Suzhou Geruisi, China). Three biological replicates and three technical replicates were used for each assay.

### Assessment of photosynthesis under salt stress

#### Chlorophyll content

Leaves were collected from each treatment, and the pigments were extracted by incubating the samples in 96% ethanol in darkness. The extracts were centrifuged, and the absorbance of the supernatant was recorded at 649 nm and 665 nm with a UV–visible spectrophotometer (Biomate 3S, Thermo Fisher Scientific, Waltham, MA, United States). The total chlorophyll content and the chlorophyll a and b concentrations were calculated using the following formulas (Ritchie, 2006):


$$\mathrm{Chl}\;a=13.95\times {\mathrm{OD}}_{665}–6.88\times {\mathrm{OD}}_{649}$$

$$\mathrm{Chl}\;b=24.96\times {\mathrm{OD}}_{649}–7.32\times {\mathrm{OD}}_{665}$$

Chlorophyll content(mg/g)=Chla+Chlb

#### Photosynthesis-related parameters

The photosynthetic parameters, including the net photosynthesis rate (Pn), stomatal conductance (Gs), intercellular CO_2_ concentration (Ci), and transpiration rate (Tr), were measured for the fully developed leaves of the 4-week-old seedlings under salt stress using a LiCor 6400XT gas exchange system (Lincoln, NE, United States). During the measurement, the leaf temperature was set at 25°C, the photosynthetic photon flux density (PPFD) at 780 μmol m^−2^ s^−1^, CO_2_ concentration at 400 μmol mol^−1^, CO_2_ mixer at 500 μmol s^−1^, and relative humidity at 40%.

### Statistical analysis

Statistical analysis was performed using the Statistical Package for Social Science software (SPSS Inc., Chicago, IL; version 18.0). Data were compared using one-way ANOVA, and the differences between the treatment means were considered statistically significant at *p* < 0.05, *p* < 0.01, or *p* < 0.001 following Tukey’s test.

## Results

### Structure and development of salt bladders in *Chenopodium album*

**Figure 1:**
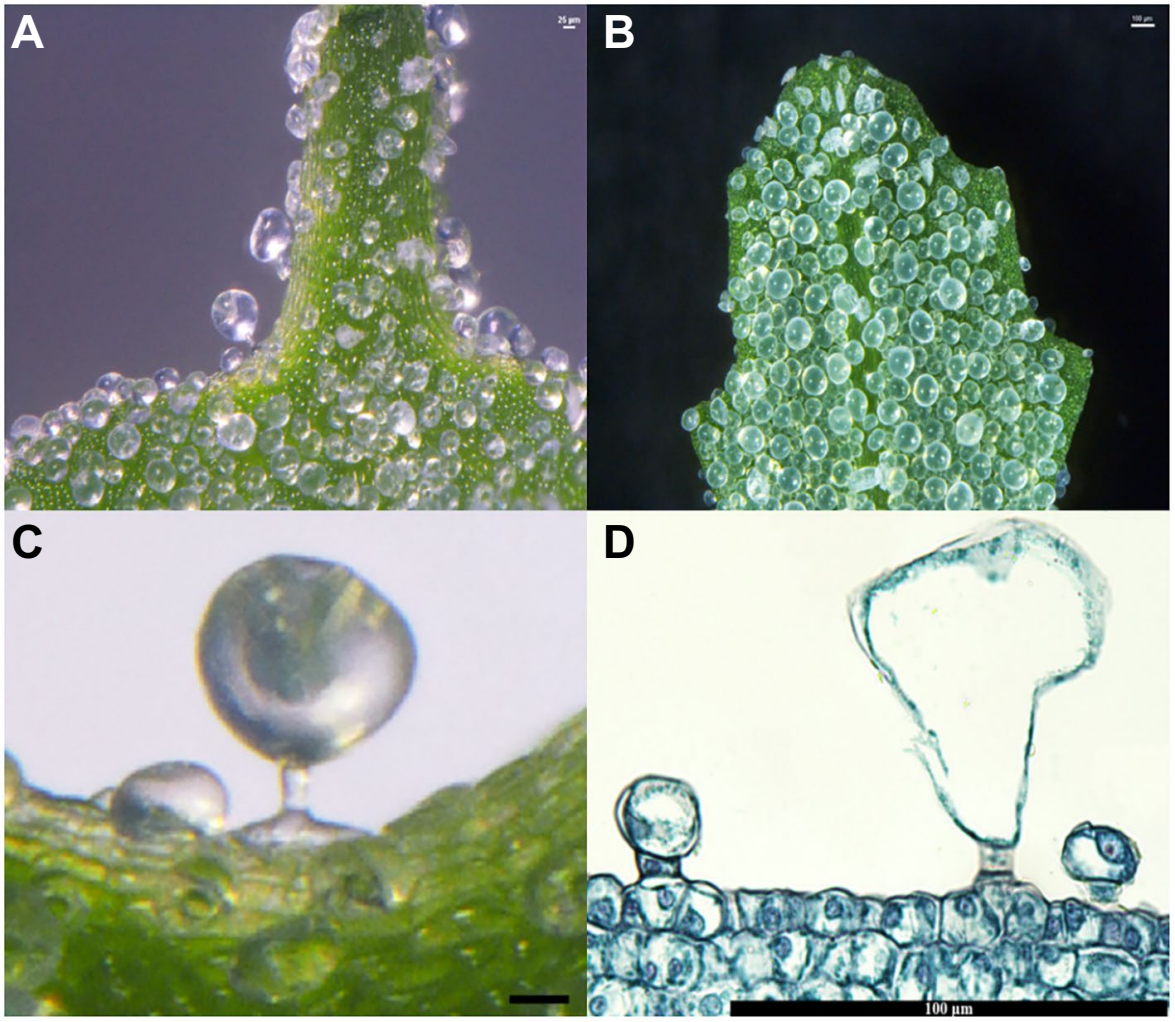
Salt bladders from *Chenopodium album*. **(A)** Salt bladders are appressed to the shoot tip surface; **(B)** Salt bladders are appressed to the young leaf surface; **(C,D)** Magnification of a single salt bladders.

The salt bladders in *C. album* were mainly found in the shoot tip and the young leaves (Figures 1A,B). Analysis of the leaf sections showed that salt bladders had one bladder cell and one stalk cell (Figure 1C). The bladder cell was huge, with a large vacuole that squeezed the nucleus to the edge of the cell (Figure 1D). The bladder cell was connected to the epidermal cells through a rectangular stalk cell with a nucleus at the center (Figures 1C,D).

**Figure 2:**
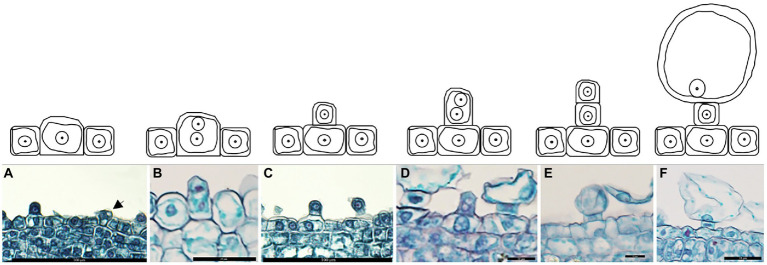
Development of salt bladder in *Chenopodium album*. **(A)** Expanded epidermal cells; **(B)** Dividing epidermal cells; **(C)** Initial bladder cell; **(D)** Dividing initial bladder cell; **(E)** Daughter cell; **(F)** Stalk cell and bladder cell.

The analysis of the salt bladder development showed that the epidermal cells on the shoot tips and young leaves generated the salt bladders. Initially, a few epidermal cells developed a dense protoplasm (presented in a darker color) that continuously enlarged (Figure 2A) and formed vertical protrusions (Figures 2B,C). These cells continued to stretch and divide to form two daughter cells (Figures 2D,E); one cell near the epidermis became the stalk cell, while the other one far from the epidermis continued to expand and developed into the bladder cell (Figure 2F). These salt bladders swelled during development and ruptured at leaf maturity due to increased internal lysate content or an external force.

### Localization of cell wall polysaccharides

**Figure 3:**
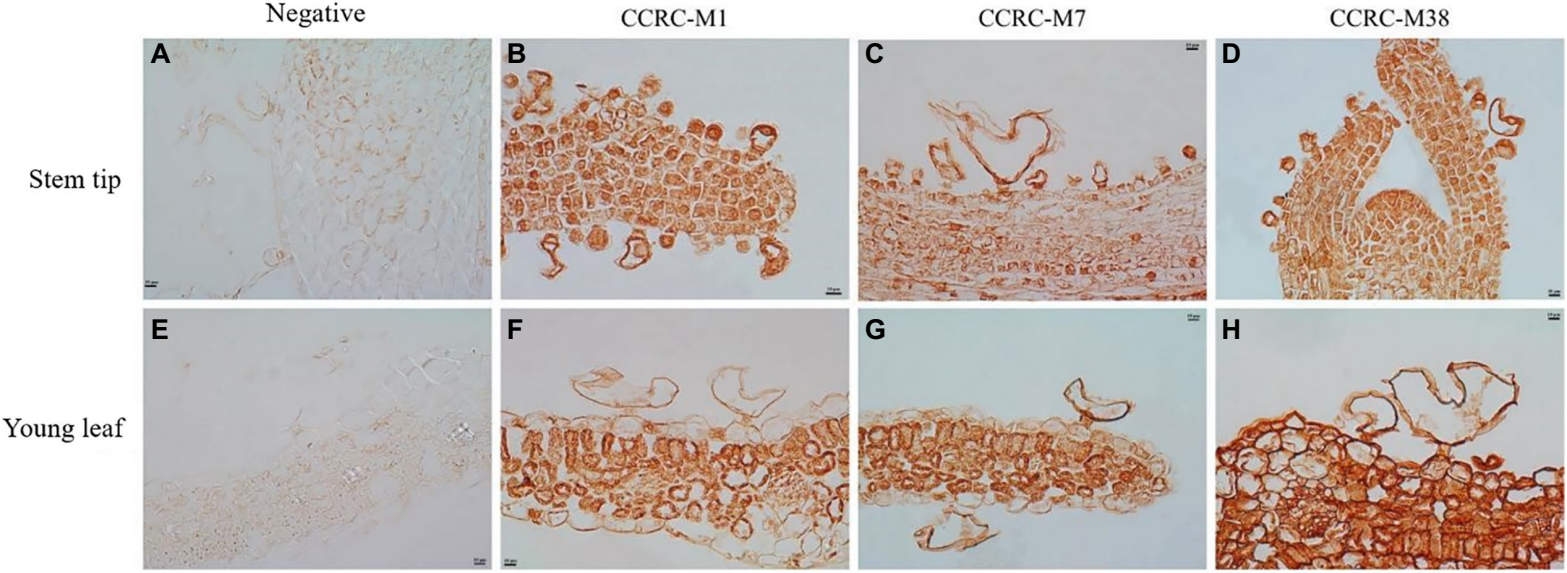
Fucose, arabinogalactan epitope, and de-esterified homogalacturonan in *Chenopodium album* stem tip and young leaf. **(A–D)** Stem tip; **(E–H)** Young leaf.

The sections of tissues from shoot tip to young leaf were probed with antibodies that recognize a diverse array of polysaccharide and glycoprotein epitopes. In this study, *in situ* immunohistochemical analysis detected no polysaccharide or glycoprotein in the control, indicating the specific action of the primary antibodies (Figures 3A,E). Meanwhile, the fucosylated epitope detected by CCRC-M1 was higher in the bladder cell wall than in the epidermal cell wall (Figure 3B), and the intensity was low in the salt bladders at young leaf (Figure 3F). These results indicated that the content of xyloglucan in cell walls gradually decreased with the development of salt bladder cells. Meanwhile, the arabinogalactan protein (AGP), recognized by the CCRC-M7 antibody, was evenly distributed in the cell wall of salt bladders at all stages of stem tip and young leaf development, and the content was higher than that of the epidermal cell wall (Figures 3C,G). The galacturonan (HG) epitope of pectin, recognized by CCRC-M38, remained a constant throughout salt bladder development; moreover, no difference was detected between the salt bladder cells and the epidermal cells (Figures 3D,H).

### Effects of salt stress on Na^+^ and K^+^ accumulation in *Chenopodium album* leaves

**Figure 4:**
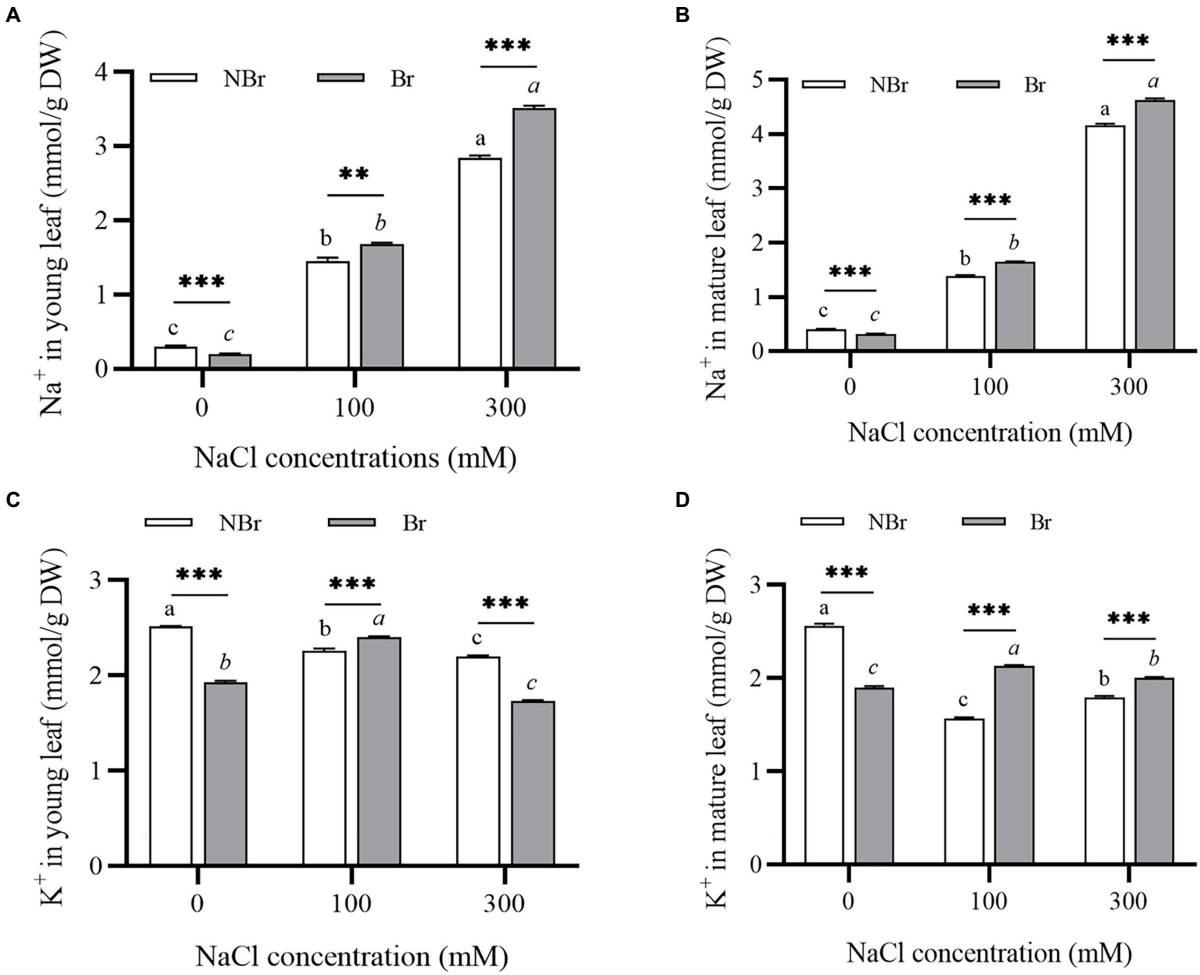
Ion contents in the leaves of *Chenopodium album* seedlings under different NaCl treatments. (**A**) Na^+^ content in young leaf; (**B**) Na^+^ content in mature leaf; (**C**) K^+^ content in young leaf; (**D**) K^+^ content in mature leaf. Different lowercase letters indicate significant differences among the treatments in the unbrushed group, and different italic lowercase letters indicate significant differences among the treatments in the brushed group. Asterisk indicates a significant difference between the brushed and unbrushed groups. ^**^p < 0.01 and ^***^p < 0.001 (Tukey’s test).

Further, to investigate the effect of salt stress on the Na^+^/K^+^ homeostasis of *C. album* leaves, the concentrations of Na^+^ and K^+^ in the young and mature leaves were measured under 100 mM or 300 mM NaCl stress. The analysis detected a significant increase in Na^+^ content in young and mature leaves of both groups under NaCl stress (Figures 4A,B). The Na^+^ content in the young and mature leaves of *C. album* with no salt bladders was substantially higher than that of the unbrushed group under control and NaCl stress. The Na^+^ content in the mature leaves of the unbrushed and brushed groups under 300 mM NaCl treatment was 10.51- and 14.76-fold higher than that under control (Figures 4A,B). On the other hand, K^+^ content under salt stress was the same as under control but significantly different between the brushed and unbrushed groups under each treatment (Figures 4C,D). This observation suggests an important role of K^+^ in regulating cation-anion balance in the leaves.

### Physiological indicators of *Chenopodium album* under salt stress

#### Osmotic regulators under salt stress

**Figure 5:**
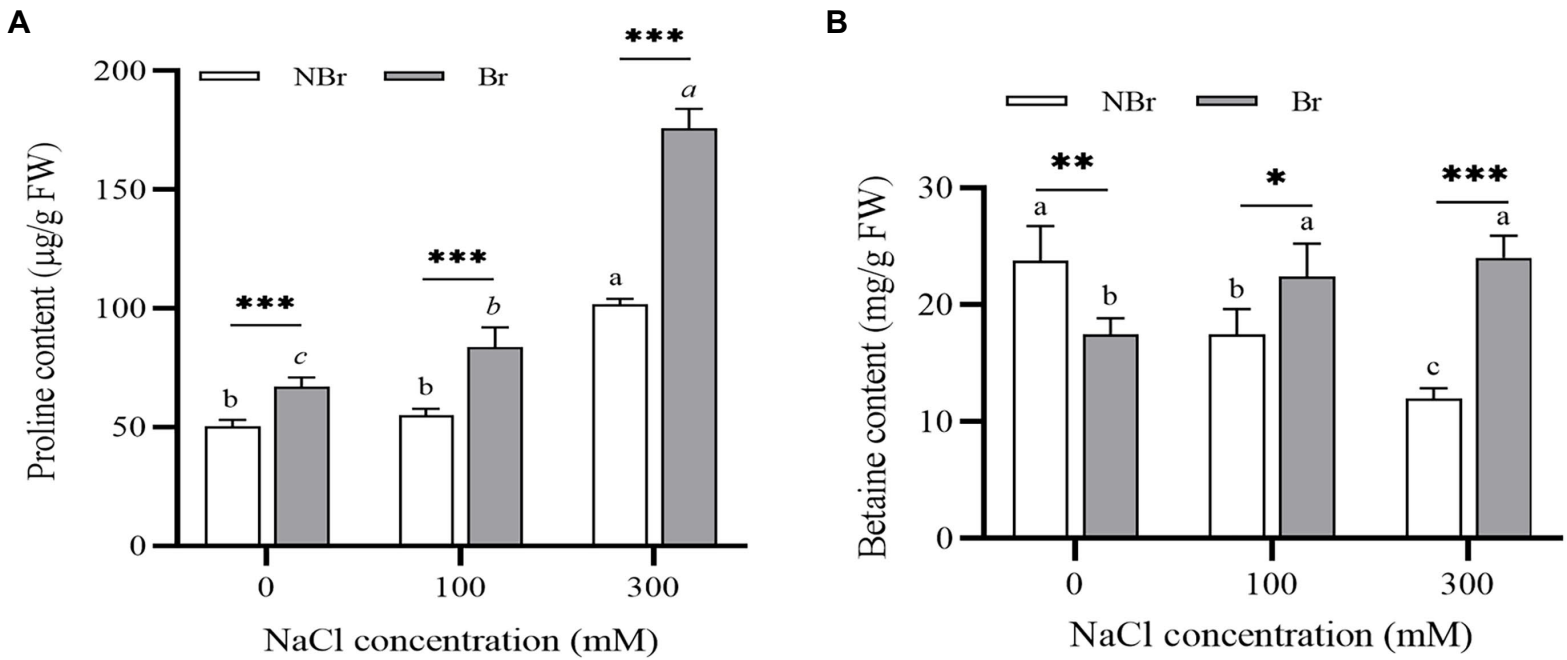
Free proline **(A)** and betaine **(B)** concentrations in the leaves of *Chenopodium album* seedlings under different NaCl treatments. Different lowercase letters indicate significant differences among the treatments in the unbrushed group, and different italic lowercase letters indicate significant differences among the treatments in the brushed group. Asterisk indicates a significant difference between the brushed and unbrushed groups. ^*^p < 0.05, ^**^p < 0.01, and ^***^p < 0.001 (Tukey’s test).

The brushed group accumulated significant amounts of osmotic regulators under salt stress compared with the unbrushed group. Under 300 mM NaCl stress, the unbrushed and the brushed groups showed an increase in proline content (2.02- and 3.63-fold, respectively) compared with the control. The proline content in the brushed group was significantly higher than that in the unbrushed group under salt stress; moreover, the proline content increased with the increase in salt concentration in both groups (Figure 5A). Meanwhile, the betaine content decreased in the unbrushed group and raised in the brushed group under salt stress; however, the brushed group had significantly higher betaine content than the unbrushed group. Under 300 mM NaCl stress, the betaine content in the brushed group was 1.99 times that of the unbrushed group (Figure 5B).

#### H_2_O_2_ and MDA content under salt stress

**Figure 6:**
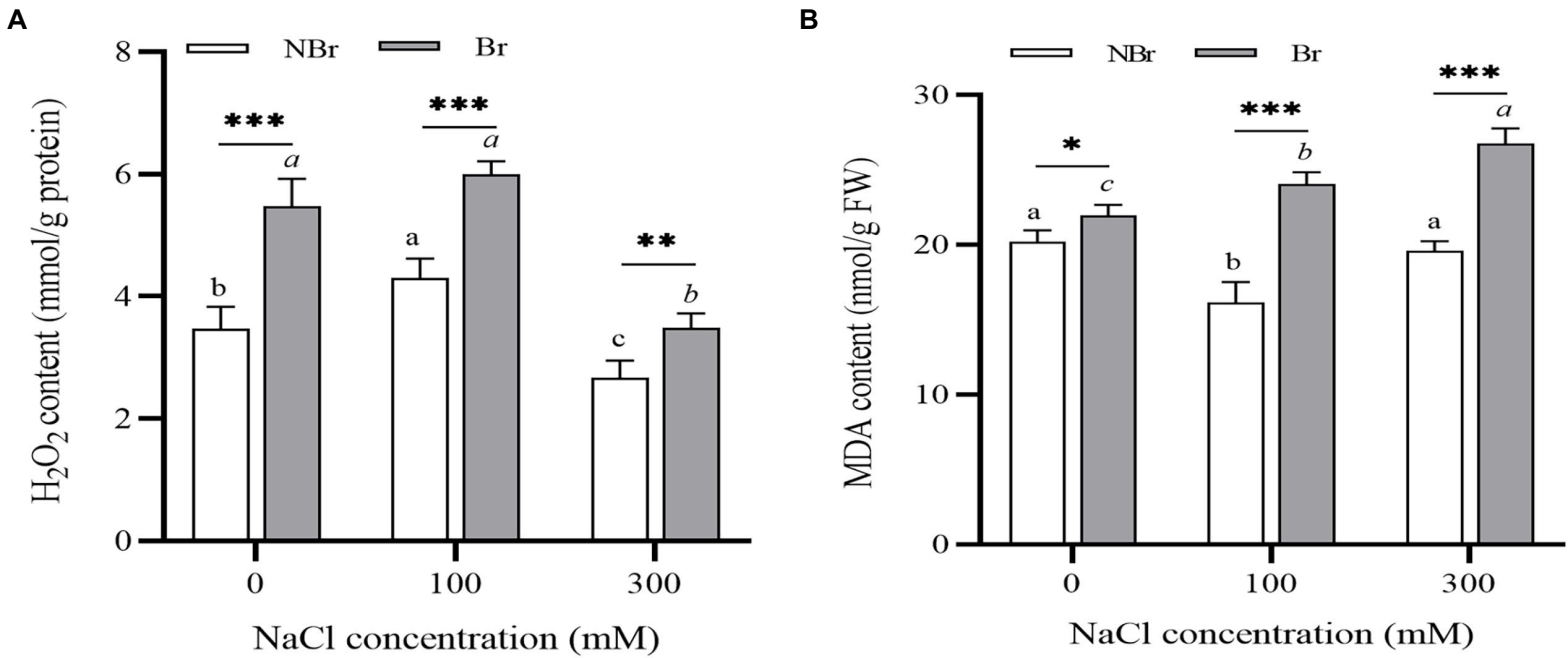
H_2_O_2_ **(A)** and MDA **(B)** concentrations in the leaves of *Chenopodium album* seedlings under different NaCl treatments. Different lowercase letters indicate significant differences among the treatments in the unbrushed group, and different italic lowercase letters indicate significant differences among the treatments in the brushed group. Asterisk indicates a significant difference between the brushed and unbrushed groups. ^*^p < 0.05, ^**^p < 0.01, and ^***^p < 0.001 (Tukey’s test).

Furthermore, we detected a decline in H_2_O_2_ content in the brushed group and the unbrushed group under high salt stress compared with the control (Figure 6A). Meanwhile, the MDA content increased in the brushed group under salt stress compared with the control, and it was significantly higher than in the unbrushed group under the control and salt stress (Figure 6B).

#### Reactive oxygen species scavenging capacity under salt stress

**Figure 7:**
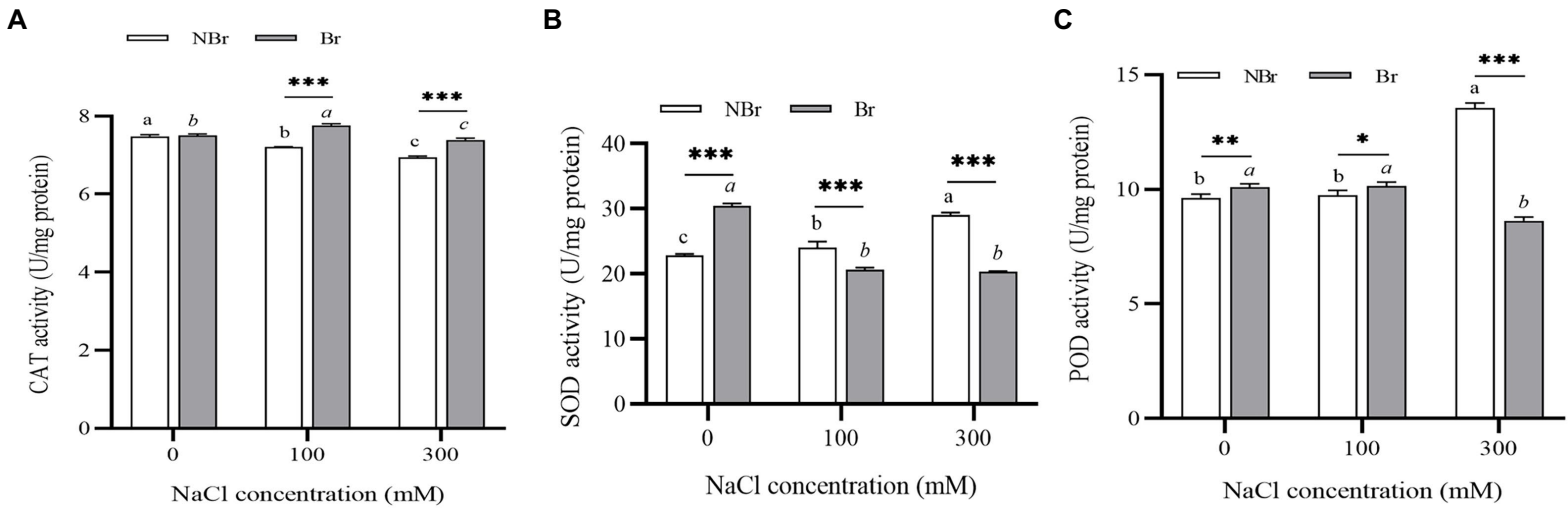
Catalase (CAT) **(A)**, superoxide dismutase (SOD) **(B)**, and peroxidase (POD) **(C)** activities in the leaves of *Chenopodium album* seedlings under different NaCl treatments. Different lowercase letters indicate significant differences among the treatments in the unbrushed group, and different italic lowercase letters indicate significant differences among the treatments in the brushed group. Asterisk indicates a significant difference between the brushed and unbrushed groups. ^*^p < 0.05, ^**^p < 0.01, and ^***^p < 0.001 (Tukey’s test).

The CAT activity of *C. album* plants in the brushed group was higher at 100 mM NaCl stress and lower at 300 mM NaCl stress than in control. In the unbrushed group, the CAT activity gradually lowered under stress. The CAT activity was significantly higher in the brushed group than in the unbrushed group under salt stress (Figure 7A). On the contrary, under control conditions, the activities of SOD and POD in the brushed group were higher than those in the unbrushed group. However, the activities in the brushed group were lower than those in the unbrushed group under high salt stress (Figures 7B,C).

### External NaCl improves the photosynthetic efficiency of *Chenopodium album*

#### Chlorophyll content under salt stress

**Figure 8:**
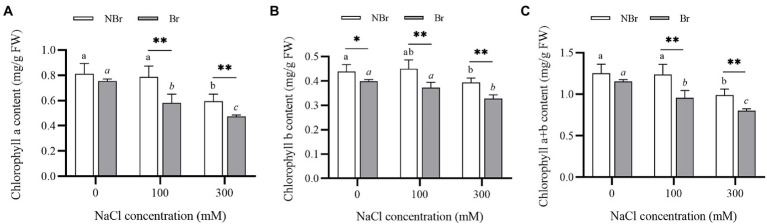
Chlorophyll a content (**A**), chlorophyll b content (**B**), and chlorophyll a+b content (**D**) in the leaves of *Chenopodium album* seedlings under different NaCl treatments. Different lowercase letters indicate significant differences among the treatments in the unbrushed group, and different italic lowercase letters indicate significant differences among the treatments in the brushed group. Asterisk indicates a significant difference between the brushed and unbrushed groups. ^*^p < 0.05 and ^**^p < 0.01 (Tukey’s test).

Salt stress can reduce the chlorophyll content of leaves, thereby affecting photosynthetic efficiency. The present study also found a decrease in the chlorophyll a, b, and total chlorophyll content with increase in salt concentration (Figures 8A,B). Moreover, the total chlorophyll content in the brushed group was significantly lower than in the unbrushed group under control or salt stress (Figure 8C).

#### Photosynthetic characteristics of *Chenopodium album* under salt stress

**Figure 9:**
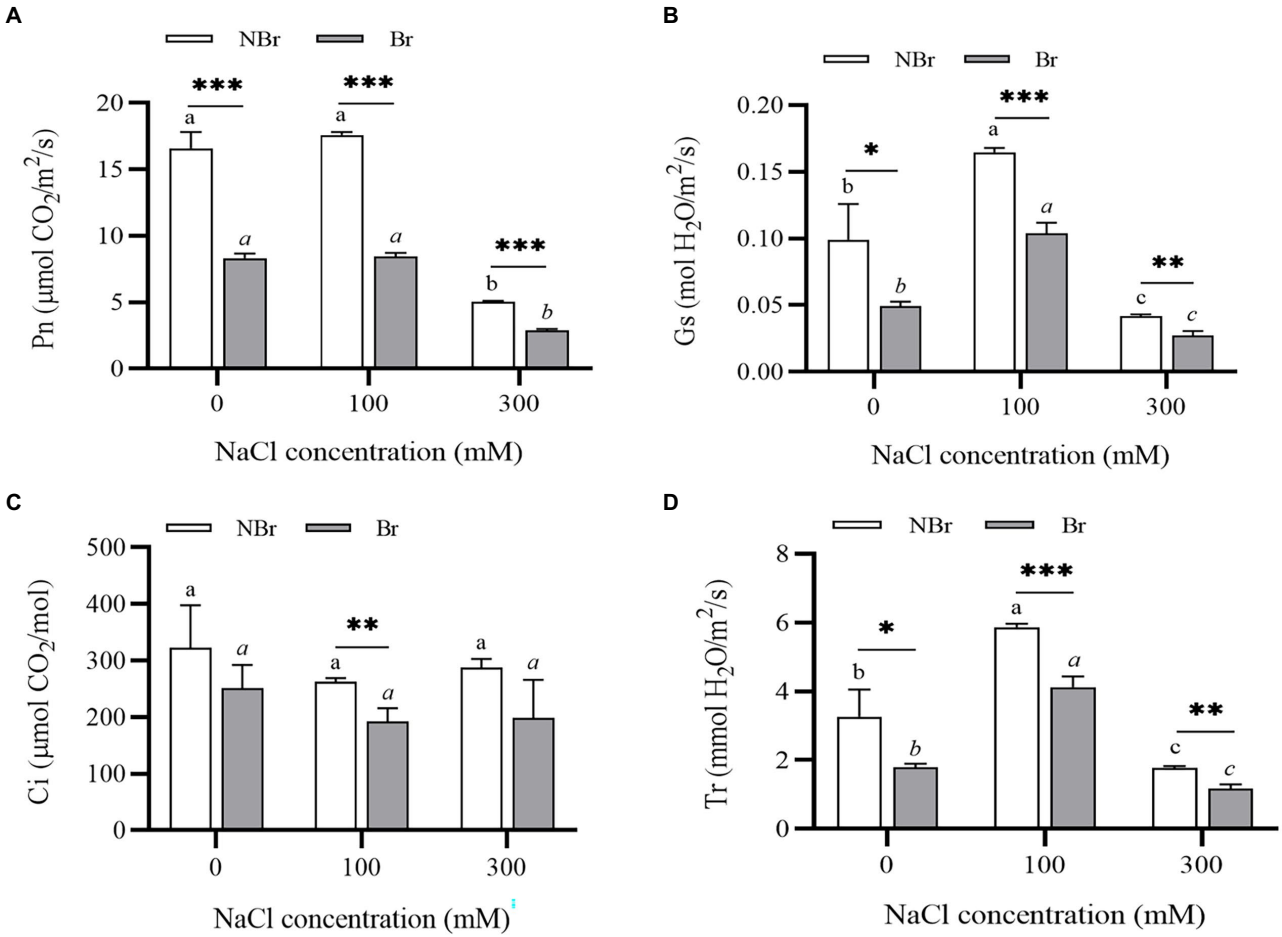
Net photosynthesis rate **(A)**, stomatal conductance **(B)**, intercellular CO_2_ concentration (C_i_) **(C)**, and transpiration rate (T) **(D)** of *Chenopodium album* seedlings under different NaCl treatments. Different lowercase letters indicate significant differences among the treatments in the unbrushed group, and different italic lowercase letters indicate significant differences among the treatments in the brushed group. Asterisk indicates a significant difference between the brushed and unbrushed groups. ^*^p < 0.05, ^**^p < 0.01, and ^***^p < 0.001 (Tukey’s test).

Likewise, we found a decrease in photosynthetic efficiency in terms of various gas exchange parameters under salt stress. However, the decrease was evident in the unbrushed group only under high salt stress (300 mM NaCl). Meanwhile, the net photosynthesis rate (Pn), stomatal conductance (Gs), intercellular CO_2_ concentration (Ci), and transpiration rate (Tr) were significantly lower in the brushed group than those of the unbrushed group under both control and salt stress (Figure 9).

## Discussion

Salt bladders are a type of specialized epidermal hairs of halophytes. These epidermal hairs’ cell structure and developmental process have been proposed in *Chenopodiaceae* (Shabala et al., 2014). Yet, the changes in the cell wall composition of salt bladders during development remain unexplored. In this experiment, we analyzed the shoot tips and young leaves of *C. album* and summarized the development of salt bladders. The results showed that the salt bladders had one bladder cell and one stalk cell in *C. album*, different from the salt bladders of other *Chenopodiaceae* species, with one or two stalk cells (Schirmer and Breckle, 1982). However, consistent with the other *Chenopodiaceae* plants, the salt bladders of *C. album* originate from the epidermal cells on the leaf surface. A few epidermal cells undergo expansion and then divide and expand, forming the bladder. We observed that only a fraction of the numerous epidermal cells on the leaf surface developed into salt bladders. Those epidermal cells forming salt bladders were usually larger than the normal cells (Figures 1, 2). Researchers have proven that the fate of an epidermal cell depends on the movement of transcription inhibitors from the differentiating cells into the neighboring cells, which possibly change the cell size (Shabala, 2013).

Under normal conditions, the plant cell wall maintains the cell structural integrity and coordinates cell division and expansion. The cell wall mainly comprises numerous polysaccharides and fewer proteins, enzymes, and fatty acids, and these components present dynamic distribution across the various stages of cell development (Samaj et al., 1999; Stenvik et al., 2006). In 1982, Schirmer and Breckle proposed that the development of the salt bladder may be closely related to the cell wall components or structures (Schirmer and Breckle, 1982); however, there was no evidence to clarify the relationship. The present study found that the content of hemicellulose xyloglucan in the cell wall of bladders at the early stage was significantly higher than that of the leaf epidermal cells (Figures 3B,F). We speculated that the high hemicellulose content might not only maintain the cell structure integrity and improve the cell wall flexibility but also contribute to the expansion and rupture of salt bladders. The detailed analysis showed that the cell wall had a higher content of pectin, which remained constant throughout the development of the salt bladders (Figures 3D,H). These ubiquitous cell wall pectin components are generally involved in developmental processes, such as cellular adhesion and stem elongation (Micheli, 2001). In *Gossypium* and *Galium aparine*, the arabinogalactan protein (AGP), a hydroxyproline-rich of glycoprotein, has been closely associated with cell division and expansion, tissue shedding, and growth signaling (Bowling et al., 2008, 2011). Moreover, a massive accumulation of AGP promoted the decomposition of xyloglucan, resulting in tissue shedding (Bowling et al., 2011). Labeling the AGP epitope in the cell wall in this study showed that AGP was abundantly distributed in the cell wall, and the content was higher in the bladder cell than that in the epidermal cells during the whole developmental process (Figures 3C,G). These findings suggest the role of AGP in the development and expansion of salt bladders in *C. album*.

In plants, salt stress hinders water absorption, leading to Na^+^ accumulation and disrupting ion homeostasis; these changes result in metabolic disorders and growth retardation (Xu et al., 2016). Meanwhile, halophytes use the specialized salt-secreting structures called salt bladders to absorb excess Na^+^ and excrete it through rupture (Shabala et al., 2014). Being a true halophyte, quinoa plants benefit from having Na^+^ in the growth media; they chelate Na^+^ mainly through salt bladders, which also accumulate 50% of K^+^ and 40% of Cl^−^ under salt stress (Kiani-Pouya et al., 2019). The present study confirmed that salt bladders absorbed a lot of Na^+^ and K^+^. However, when the salt bladders were removed, numerous Na^+^ were detected in the mesophyll cells under salt stress (Figures 4A,B). In addition, the K^+^ content in the leaves of the brushed group decreased under salt stress, and the content was significantly lower than that of the unbrushed group (Figure 4C). Therefore, we speculate that salt stress destroyed the structure of cell membranes and caused intracellular ion leakage; however, K^+^ played a key role in maintaining intracellular ion balance and osmotic pressure stability. Thus, retaining K^+^ and maintaining Na^+^/K^+^ homeostasis is important for salt tolerance (Shabala and Cuin, 2008; Hariadi et al., 2010). These results are consistent with the reports in *Chenopodium quinoa* Willd. (Kiani-Pouya et al., 2017) and *Atriplex canescens* (Guo et al., 2019) and confirm that salt bladders increase the leaf K^+^ retention capacity and improve salt tolerance.

Under abiotic stress, plants accumulate osmotic regulators that maintain cell osmotic pressure, protect cell and protein integrity, and prevent cells from oxidative damage by scavenging reactive oxygen species (Szabados and Savouré, 2010; Uzilday et al., 2015). These changes reduce the adverse effects of abiotic stress on plants. In this experiment, the leaf proline and betaine content increased significantly in the brushed group under salt stress (Figure 5). Thus, the plants without salt bladders were more susceptible to ionic damage and had to deal with stress by accumulating proline. Under high NaCl treatment, CAT activity was higher in the brushed plants than in the unbrushed ones (Figure 7A), while the SOD and POD activities were significantly downregulated in the brushed plants (Figures 7B,C). These observations suggest that the plants without salt bladder (brushing) significantly increased reactive oxygen species scavenging through CAT activity; however, they did not get damaged by SOD and POD activities under NaCl treatment.

In addition, we found significantly low leaf chlorophyll content in the brushed plants (Figure 8). The Pn, Gs, Ci, and Tr were substantially lower in the brushed group than in the unbrushed group under control and stress conditions (Figure 9). This observation is consistent with quinoa (Böhm et al., 2018) and indicates that the salt bladder of *C. album* contained many chloroplasts. Therefore, brushing reduced photosynthesis even under normal conditions. Moreover, the brushed plants had significantly low chlorophyll content and photosynthetic efficiency and were more sensitive to salt stress.

## Conclusion

The present study found salt bladders on the young leaves of the desert plant *C. album* that ruptured and fell off with the development of leaves. Analysis of the salt bladder cell wall revealed variations in xyloglucan and AGP content during development. Salt bladders played a crucial role in salt tolerance by protecting young leaves and improving photosynthetic efficiency. However, the mechanisms *via* which the salt bladders initiate development, regulate volume, rupture, and shedding, and modulate the associated genes remain unexplored. Thus, the findings provide an important reference for further research on salt bladders in engineering salt-tolerant plants.

## Data availability statement

The original contributions presented in the study are included in the article/supplementary material, further inquiries can be directed to the corresponding author.

## Author contributions

YZ and HL designed the experiment and methodology. AM carried out experimental work. YZ wrote the manuscript. All authors contributed to experimental design and data analysis, commented on the manuscript, and gave final approval for publication.

## Funding

This work was supported by the Science and Technology Training Project for Excellent Young Scholars of Xinjiang (no. 2019Q014) and National Natural Science Foundation of China (no. 31900270).

## Conflict of interest

The authors declare that the research was conducted in the absence of any commercial or financial relationships that could be construed as a potential conflict of interest.

## Publisher’s note

All claims expressed in this article are solely those of the authors and do not necessarily represent those of their affiliated organizations, or those of the publisher, the editors and the reviewers. Any product that may be evaluated in this article, or claim that may be made by its manufacturer, is not guaranteed or endorsed by the publisher.
